# Estimating the Shear Resistance of Flocculated Kaolin Aggregates: Effect of Flocculation Time, Flocculant Dose, and Water Quality

**DOI:** 10.3390/polym14071381

**Published:** 2022-03-29

**Authors:** Kevin Pérez, Norman Toro, Matías Jeldres, Edelmira Gálvez, Pedro Robles, Omar Alvarado, Pedro G. Toledo, Ricardo I. Jeldres

**Affiliations:** 1 Departamento de Ingeniería Química y Procesos de Minerales, Facultad de Ingeniería, Universidad de Antofagasta, Av. Angamos 601, Antofagasta 1240000, Chile; kevin.perez.salinas@ua.cl (K.P.); hugo.jeldres.valenzuela@ua.cl (M.J.); 2 Faculty of Engineering and Architecture, Universidad Arturo Prat, Almirante Juan José Latorre 2901, Antofagasta 1244260, Chile; notoro@unap.cl; 3 Department of Metallurgical and Mining Engineering, North Catholic University, Angamos Av. 0610, Antofagasta 1270709, Chile; egalvez@ucn.cl; 4 Escuela de Ingeniería Química, Pontificia Universidad Católica de Valparaíso, Valparaíso 2340000, Chile; pedro.robles@pucv.cl; 5 Departamento de Química, Facultad de Ciencias, Universidad del Bio Bio, Av. Collao 1202, Concepción 4030000, Chile; oalvarado@ubiobio.cl; 6 Department of Chemical Engineering and Laboratory of Surface Analysis (ASIF), Universidad de Concepcion, P.O. Box 160-C, Correo 3, Concepción 4030000, Chile; petoledo@udec.cl

**Keywords:** kaolin flocculation, aggregate resistance, salinity, flocculation kinetic, shear rate

## Abstract

The resistance of kaolin aggregates to shearing in water clarification and recovery operations is a critical input in designing thickener feed wells. A recently formulated but already available criterion is used to determine the shear strength of flocculated kaolin aggregates. The flocculant is a high molecular weight anionic polyelectrolyte. The resistance of the aggregates is evaluated as a function of flocculation time, flocculant dosage, and water quality. The determination is based on a standardized experimental method. First, the time evolution of the average size of kaolin flocs is measured when aggregates are exposed to incremental shear rates from a predetermined base value. Then, the results are fitted to a pseudo-first-order model that allows deriving a characteristic value of the shear rate of rupture associated with the upper limit of the strength of the aggregates. In seawater, at a given dose of flocculant, the strength of the aggregates increases with time up to a maximum; however, at longer times, the resistance decreases until it settles at a stable value corresponding to stable aggregates in size and structure. A higher flocculant dosage leads to stronger aggregates due to more bridges between particles and polymers, leading to a more intricate and resistant particle network. In industrial water with very low salt content, the resistance of the kaolin aggregates is higher than in seawater for the same dose of flocculant. The salt weakens the resistance of the aggregates and works against the efficiency of the flocculant. The study should be of practical interest to concentration plants that use seawater in their operations.

## 1. Introduction

The separation of solids from liquids in a thickener is driven by gravity leading to a dense slurry containing most solids under a zone of clarified liquid. The dense slurry is swept into the thickener discharge by rotating rakes from where it is transported to tailings deposits. The clarified liquid is the thickener overflow that is recirculated to the process. The liquid is generally water, which is recovered by thickening through sedimentation applied to product and tailings streams. When the particle sizes are small, <80 µm, the gravitational effects are negligible, and the sedimentation is poor. Thus, the application of water-soluble polymers is key to the formation of massive aggregates that accelerate the rate of sedimentation. The most widely used reagents to destabilize tailings particles and aggregates are high molecular weight anionic polyacrylamides (A-PAM) due to high sedimentation rates that are achieved at relatively low doses and costs [1,2].

Flocculation performance is susceptible to shear applied in the thickener feedwell. A low shear rate limits the dispersion and contact between particles and flocculant chains. Therefore, the flocs formed do not reach an adequate settlement. At the same time, an excessive shear causes the detachment of the polymer chains from the particles and breakage of particle–flocculant–particle bonds, impacting the size and structure of the aggregates. Heath et al. [3] used a population balance (PB) model to describe the aggregation/breakage kinetics in calcite flocculation in turbulent pipe flow, demonstrating that the aggregates decrease in size once a maximum size is reached due to fragmentation by shear and polymer degradation, both irreversible events.

Their size and structure characterize aggregates through properties such as size distribution, strength, and fractal dimension. These properties directly impact tailings management indicators such as the water recovery rate, the quality of the recovered water, and the rheological properties of the thickened slurries. The relationship between the mass *m* in a sphere of radius *R* of a self-similar fractal aggregate obeys $m\propto R^{D_{f}}$, with $D_{f}$ the fractal dimension [4]. In a space of *d* Euclidean dimensions $D_{f}\leq d$, and if d=3 then $1\leq D_{f}\leq 3$. The largest values of $D_{f}$ correspond to dense and resistant spherical flocs, whereas the smallest ones correspond to ultra-light and brittle rod-like aggregates. The prefactor represents the structure of the aggregate; however, it is not generally considered even though it is needed in conjunction with $D_{f}$ to determine the mass of an aggregate from its size.

The fractal dimension of aggregates can be estimated using image analysis and light scattering techniques and hindered sedimentation models [5,6,7]. Image analysis is the oldest and most popular technique despite the immense data processing load involved and the resolution that may be insufficient for compact fractals [6]. Light scattering is the most appropriate technique if the flocs are small, optically translucent, and diluted. The technique has been used successfully in colloidal systems [8,9,10,11] but not in aggregates with high clay content. Estimates of $D_{f}$ from hindered sedimentation data require the average size of the aggregates, which may be non-trivial if the slurry is strongly sheared before sedimentation because then the model can yield fractal dimensions that decrease with agitation [12]. The question is whether the aggregates are effectively no longer self-similar or the size definition should be adjusted for highly-sheared aggregates. The advantage of this last methodology is that it provides structural properties of aggregates under conditions that mimic flocculation in the feedwell of industrial thickeners.

Deng and Davè [13] analyzed the effect of impact velocity and surface energies on the structure of spherical particle agglomerates using the discrete element method (DEM). The authors found a significant reduction in the fractal dimension with increasing shear, stating that they grow irregularly when the aggregates are produced with a greater mixing intensity. Additionally, they consider that aggressive hydrodynamics deteriorates the structure of the aggregates, which promotes the reduction of the fractal dimension. Finally, Leiva et al. [12] analyzed the impact of flocculation time and shear on the structural characteristics of HPAM-flocculated clay tailings aggregates in seawater. The authors found that the more an aggregate is exposed to hydrodynamic disturbances, the more significant the deterioration of its structure, which is reflected in lower fractal dimension values.

The structural properties of the flocs are determined by the characteristics of the mineral, mineralogy, and size distribution; characteristics of the flocculant, structure, molecular weight, charge density and dosage; conditions of the environment, salinity, and pH; and shear rate during mixing [14,15,16]. Although various studies relate the control variables in the feedwell of a thickener with the properties of the aggregates and the flocculation performance [17,18,19,20], few do so when the medium is seawater. Using molecular dynamics, Quezada et al. [21] studied the impact of salinity on the conformation of an anionic polyelectrolyte on quartz surfaces. These authors demonstrated that increasing the salinity improves the adsorption of polyelectrolytes on quartz surfaces. However, at the same time, it reduces the radius of gyration of the polyelectrolyte, limiting the size of the aggregation achieved. Jeldres et al. [22] carried out flocculation and sedimentation experiments on suspensions of quartz and kaolinite mixtures, finding a direct relationship between flocs microscopic properties, fractal dimension, and some macroscopic observables such as sedimentation speed and yield stress of the sediment. Flocs with a larger fractal dimension settled at higher speeds, implying an increase in the yield stress of the sediment. This last parameter presented an exponential growth with the fractal dimension for all the shear conditions studied. The authors explain this behavior because all three parameters are dependent on both the size and structure of the aggregate.

Floc resistance is directly related to the number and strength of interparticle bonds [23,24]; it is considered a key parameter in solid/liquid separation since it determines the degree of floc breakage. A floc breaks if the applied stress is greater than the force that holds the particles together. Operationally, this is important since the small particles of a fragmented floc have a lower sedimentation rate. However, a high floc resistance could increase the sediment’s rheological properties, which implies a higher energy consumption for its transport to the tailings deposit. Therefore, the degree of resistance must remain in a range that guarantees industrially acceptable rates of sedimentation and magnitudes of rheological properties.

Recently, Jeldres et al. [25] proposed a simple criterion to quantitatively estimate the strength of aggregates subjected to increasing mechanical shear disturbances. The monotonic relationship between the shear rate increments (Δ*G*) and the final size of the aggregates is used for a more quantitative estimate of the resistance of the target aggregates since a pseudo-first-order model can describe this relationship. The main result is a parameter that does not depend on the hydrodynamic conditions chosen to establish the disturbances unlike models available in the literature, which, although they work adequately within the experimental conditions carried out for their validation, are not necessarily extrapolated and comparable in different systems. The methodology was used to evaluate the impact of salinity on the resistance of clay aggregates. The results show that floc breakage is achieved with considerably lower agitation increments as the salinity of the system increases.

In this work, the resistance of kaolin flocs is related to its structural properties characterized by the fractal dimension. The kaolin suspension is flocculated with a high molecular weight anionic polyelectrolyte. The objective is to compare the strength of the aggregates and their fractal dimension both in seawater and in industrial water with low ionic charge. Additionally, the effect of the flocculation time is studied, being one of the variables handled in the thickeners’ feedwell. Kaolinite is a typical clay in tailings of copper mining operations. The particles have a colloidal size and platelet shape that represent major challenges in thickening. The challenges relate to a decrease in sedimentation rate [26,27] and an increase in rheological parameters [28,29], which are closely related to the mechanical resistance of the suspension aggregate structure. The study is of practical interest to the copper industry, especially for seawater plants in their operations. The demand for seawater has been increasing in recent years [30,31,32] as a result of the search for alternatives to face the scarcity of water resources due to climate change.

## 2. Materials and Methods

### 2.1. Materials

Seawater from Bahía San Jorge in Antofagasta (Chile) was filtered at 1 μm using a UV filter system to remove all bacterial activity. The conductivity was 50.4 mS/cm. The ionic composition and the methods used are specified in Table 1. Industrial water was prepared with 0.01 M analytical grade NaCl from Merck (Santiago, Chile).

**Figure 1:**
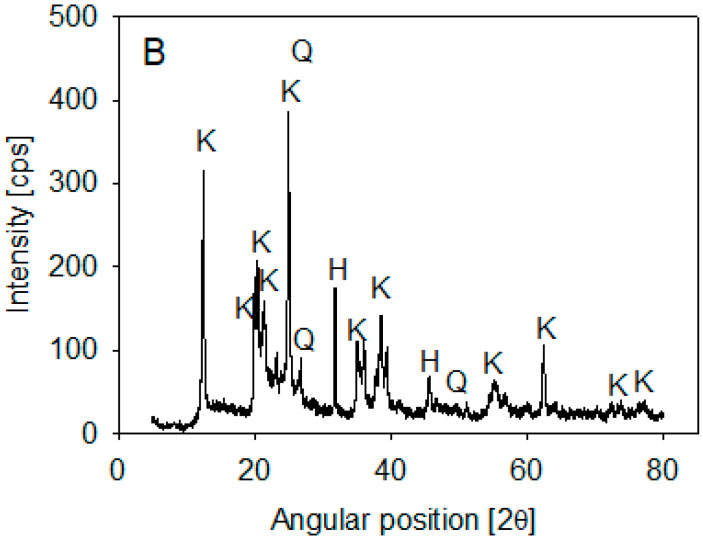
XRD of kaolin particles.

Ward’s Science kaolin particles were used. Its composition was determined by XRD analysis on a Bruker X-ray diffractometer, D8 Advance (Bruker, Billerica, MA, USA), using the 2020 ICDD (International Center for Diffraction Data) Powder Diffraction File database. The spectra showed that kaolin is mainly composed of halite and kaolinite (>10%) and some SiO2 (1–10%) (Figure 1). Volume-weighted particle size distribution (PSD) was obtained using a Microtrac S3500 laser diffraction analyzer (Verder Scientific, Newtown, PA, USA) and published in Jeldres et al. [25]. The data showed that 10% of the kaolin particles were smaller than 1.8 µm.

The flocculant used was commercial polyacrylamide SNF704, an anionic acrylate copolymer supplied by SNF-Chile with 98% purity. NaOH was used to reach pH 8.

### 2.2. Aggregate Characterization

The flocculation of kaolin particles was carried out in a cylindrical container with a diameter of 100 mm and a capacity of 1 L. An 80 mm diameter turbine impeller, attached to the end of a vertical shaft (4 mm diameter), was used to keep the particles suspended. The impeller base was located 20 mm above the bottom of the vessel.

The minerals were mixed with water (seawater and industrial water with 0.01 M NaCl) to give a total mass of 270 g. The mixture was stirred at 600 rpm (*G* = 1400 s^−1^ approximately) for 30 min at pH 8 to keep the particles dispersed before flocculation. Stirring was then reduced to 200 rpm (*G* = 273 s^−1^). After 1 min, the flocculant was added in doses of 8 to 89 g/t. The evolution of the average size of flocs was analyzed for 6 min. The strength of the aggregates was evaluated by increasing the mixing intensity. For this, the flocculation tests were repeated at the standard agitation of 200 rpm, but after a determined flocculation time, which was considered to be in the range of 30–120 s, the mixing intensity was increased to promote shear fragmentation. Independent tests were performed by applying shear rate increments from the initial *G* = 273 s^−1^ (0 to 1516 s^−1^).

The Focused Beam Reflectance Measurement (FBRM) system (Particle Track E25, Mettler Toledo, OH, USA) was used to measure the aggregate size evolution. The system consists of a processing unit with a 19 mm diameter probe tip positioned vertically in the flocculation vessel 10 mm above the stirrer and 20 mm off the axis of rotation. The technology is based on the backscattering of light using a laser beam rotating at 2 m/s and projected onto the sample suspension through a sapphire window (14 mm in diameter) at the end of the probe. The chord length, which correlates with the actual size of the particles, is obtained by multiplying the light backscatter time of each particle by the scanning speed. The instrument’s software allows recording data processing into histograms of counts corresponding to chord lengths in selected channel sizes ranging from 1 μm to 1 mm as quickly as every 2 s. In this case, the chord length distributions (CLDs) represent 100 channels over the entire range, but the histograms are presented as line plots for easy comparison. The equipment offers two types of distributions, the unweighted CLD that provides enhanced resolution to fine particle changes and the squared-weighted CLD that provides enhanced resolution to coarse particle changes. The former is useful in studies of primary nucleation, particle growth, and aggregation, and the latter is useful in studies of breakage, attrition, secondary nucleation, and particle dissolution. The average size of the aggregates was obtained considering the squared-weighted chord distribution, which has been documented to be more similar to the distribution obtained with laser diffraction.

### 2.3. Shear Rate

The mean shear rate required by the aggregation and breakage of flocs was calculated from:

(1) $$G={\left(\frac{\varepsilon \rho_{sus}}{\mu_{sus}}\right)}^{1/2}$$

where $\mu_{sus}$ is the viscosity (4 mPas), $\rho_{sus}$ is the pulp density, and *ε* is the average energy dissipation rate:

(2) $$\varepsilon =\frac{N_{p}N^{3}D^{5}}{V}$$

$N_{p}$ is the impeller power number (0.6 in our case for a plane disk with gentle agitation [2]), *N* is the rotation speed, *D* and *V* are, respectively, the diameter of the impeller and the working volume of the impeller vessel. The density of the suspension $\rho_{sus}$ was calculated from:

(3) $$\rho_{sus}={\left(\frac{w}{\rho_{s}}+\frac{1-w}{\rho_{w}}\right)}^{-1}$$

where *w* is the mass fraction of solids in suspension, and $\rho_{s}$ and $\rho_{w}$ are the density of solid and water, respectively. The parameters are indicated in Table 2.

### 2.4. Sedimentation Tests

The setting rate was determined by interrupting the flocculation tests at specific preset times, 10, 60, 90, and 120 s. The resulting suspensions were gently poured into 300 cm^3^ cylinders (35 mm internal diameter). Then, each cylinder with its content was slowly inverted three times (each cylinder inversion took ~4 s) and then placed on a flat surface to determine the sedimentation rate classically (the change in the liquid–solid interface was recorded over 10 min).

### 2.5. Fractal Dimension

The fractal dimension $D_{f}$ of the aggregates was calculated from the model of Heath et al. [3], for the hindered settlement rate as a function of aggregate size, as

(4) $$U_{h}=\frac{\bar{d_{agg}^{2}}g\left(\rho_{s}-\rho_{l}\right){\left(\frac{\bar{d_{agg}}}{\bar{d_{p}}}\right)}^{D_{f}-3}}{18\mu }{\left(1-\varphi_{s}{\left(\frac{d_{agg}}{d_{p}}\right)}^{3-D_{f}}\right)}^{4.65}$$

where $U_{h}$ is the hindered settling rate in m/s, *d_p_* is the average size of primitive particles, *d_agg_* is the average size of aggregates after some flocculation time, in this work both *d_p_* and *d_agg_* are approximated by the squared-weighted mean chord length, *ρ_s_* and *ρ_l_* are, respectively, the densities of solid and liquid phases, *g* is the acceleration of gravity, *μ* is the fluid viscosity, $\varphi_{s}$ is the solid fraction, and *D_f_* is the mass fractal dimension. Many of these parameters remain constant across experiments, for example, *ρ_s_*, *ρ_l_*, *μ*, $\varphi_{s}$, and *d_p_*. While the hindered sedimentation rate was experimentally determined after flocculation as described above. $D_{f}$ was calculated from Equation (4) using the standard method of least squares.

## 3. Results

### 3.1. Aggregation with Flocculant

**Figure 2:**
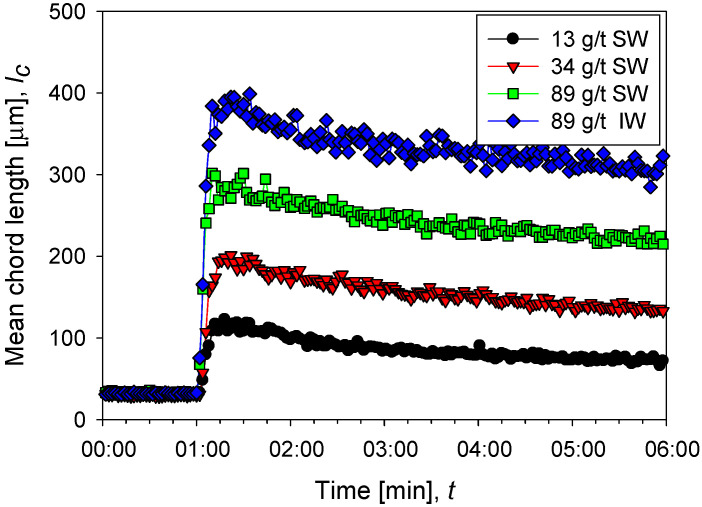
Evolution of the average size of aggregates of kaolin particles (chord length) before and after the addition of flocculant in different doses in seawater (SW) and industrial water (IW).

**Figure 3:**
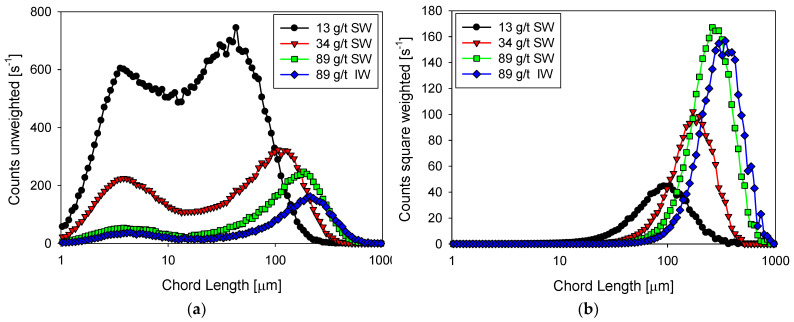
Unweighted (**a**) and squared-weighted (**b**) size distribution of kaolin suspensions with different doses of flocculant in SW and IW after three minutes of flocculation at 200 rpm immediately before increasing shear rate.

Figure 2 shows the flocculation kinetics of clay suspensions in seawater (high salt) at polyacrylamide doses of 13, 34, and 89 g/t and industrial water (low salt) at 89 g/t at a shear rate of G= 273 s^−1^. Once the flocculant is added, the growth of the aggregates is practically instantaneous, and it only takes a few seconds to reach a maximum size. Subsequently, shear fragmentation causes a continuous reduction in the size of the aggregates until reaching a stable size. The average size of aggregates shows an increasing trend with the doses studied; for example, at 13 g/t in seawater, a size peak of 115 µm is reached, while at 89 g/t the peak is at 300 µm. A saline system can favor or harm flocculation. The final result depends on the competition of two mechanisms propitiated by the presence of cations, that is, (i) better flocculant adsorption via cationic bridges and (ii) electrostatic shielding of the active sites in the flocculant, leading to polymer curling. The results in Figure 2 show better flocculation in industrial water than in seawater, indicating that the high concentration of cations in seawater causes a significant increase in polymer curling, limiting its ability to form hydrogen bonds and thus its effectiveness as a flocculant. Figure 3 shows unweighted and squared-weighted chord length distributions (CLD) for the kaolin suspension at 4 wt%, using seawater at polyacrylamide doses of 13, 34, and 89 g/t and industrial water at 89 g/t. The chosen mixing time is 55 s, just before applying an increase in shear rate.

In seawater and as the flocculant dose increases, the squared weighted distribution shows that the particles form larger and larger aggregates due to a combined process of growth (which does not change the number of particles) and agglomeration (which reduces the number of particles). The size peak with 13 g/t of flocculant occurs at 100 µm with a count of just over 40 s^−1^, while with 89 g/t the peak occurs at 200 µm with a frequency of just over 160 s^−1^. The unweighted distribution is illustrative of the particle growth process when the flocculant dose increases. At any of the flocculant doses tested, the distribution is markedly bimodal. At 13 g/t of flocculant, the distribution shows similar size frequencies of fine and coarse particles, centered, respectively, at 4 and 40 µm. The flocculant captures only a few fines at this low dosage to form larger aggregates. At the highest rate of 34 g/t, the flocculant can capture many fines (about 4 µm) to form aggregates of just over 100 µm. Then, the frequency of fine particles drops dramatically to just under 300 s^−1^. The frequency of coarse agglomerates also decreases to about 300 s^−1^. The coarse ones consume fines, and the more they consume, the lower their number. At the highest dose tested of 89 g/t, the effectiveness of the flocculant is very high because it captures practically all the fines, whose frequency decreases to less than 50 s^−1^, forming a few agglomerates that reach a peak size of 110 µm although with the lowest frequency, just over 200 s^−1^. The coiling effect of the polymer chains at high salt in seawater ceases to be a limiting factor for flocculation when the flocculant dose is high enough.

In industrial water with very low salt content and at the highest flocculant dosage of 89 g/t, the unweighted and square weighted distributions show little difference from the distributions in seawater. The flocculant forms fewer aggregates in industrial water, although larger than in seawater. The aggregate frequency in industrial water is only 150 s^−1^ (vs. 250 s^−1^ in seawater) with a peak size of 115 µm (vs. 110 µm in seawater). This result reveals that the polymer coiling effect in industrial water is less than in seawater. This effect on flocculation may be more dominant than the salt bridges that are more frequent in seawater.

### 3.2. Settling Rate

The sedimentation rate in seawater increases rapidly as the flocculant dose increases from 13 g/t to 89 g/t. Remarkable is that at low doses, 13 and 34 g/t, the rate does not change significantly and remains relatively constant as a function of mixing time. However, at 89 g/t the rate is more than double at low doses. This result is consistent with the flocculation kinetics shown in Figure 2. At higher doses, larger flocs are obtained, and therefore the population of fines is reduced (Figure 3), thus increasing the sedimentation rate. At the high flocculant dose of 89 g/t, the sedimentation rate shows a non-monotonic behavior as a function of the mixing time. At 30 s, a smooth but distinguishable maximum is observed, then the rate decreases with mixing time.

**Figure 4:**
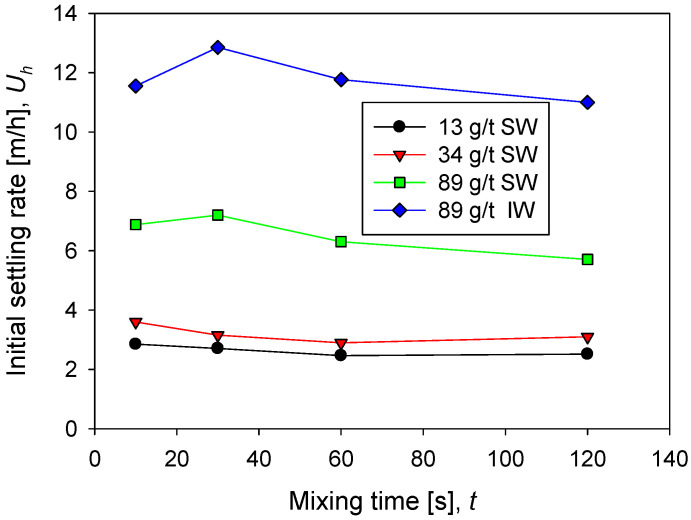
Sedimentation rate of kaolin suspensions with different doses of flocculant in SW and IW at a shear rate of 200 rpm.

The sedimentation rate in industrial water is notoriously higher than in seawater, as shown in Figure 4 at 89 g/t of flocculant in both cases. This result is also consistent with the flocculation kinetics in Figure 2, which show aggregates with larger average sizes in industrial water. The particle size distributions in Figure 4 show that coarser aggregates are larger in industrial water than in seawater. In industrial water, the sedimentation rate shows a more pronounced maximum at 30 s.

### 3.3. Fractal Dimension

The aggregation of particles in the presence of flocculant occurs through hydrogen bonds and salt bridges if the medium is saline, leading to highly irregular porous structures that cannot be described by impermeable sphere models [33,34]. There is consensus that the structure of flocculated aggregates follows a scale-invariant growth that generates fractal structures [35]. The fractal dimension directly relates to macroscopic observables of industrial interest such as rheological and settling [22].

**Figure 5:**
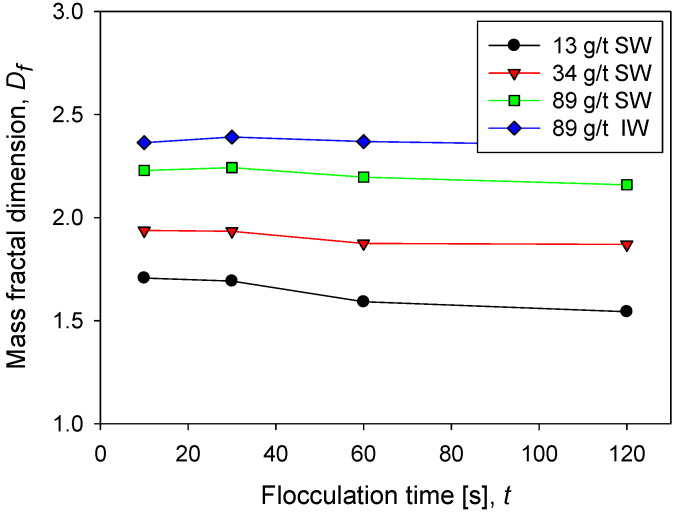
Fractal dimension of kaolin suspensions with different doses of flocculant in SW and IW at a shear rate of 200 rpm.

Figure 5 shows the behavior of the fractal dimension of kaolin aggregates as a function of the flocculation time at different doses of flocculant in seawater and in industrial water at the same shear rate. The fractal dimension of kaolin aggregates is not very sensitive to flocculation time; only at low doses of flocculant in seawater is a small decrease associated with a slight restructuring of the aggregates observed. This result is different in aggregates that come from mineral mixtures. For example, the fractal dimension of kaolin and quartz aggregates decreases with flocculation time, according to Leiva et al. [12]. At low flocculant doses in seawater, the flocs that form are small according to the flocculation kinetics (Figure 2) and the size distribution (Figure 3). Now according to their low fractal dimension (between 1.5 and 2), it is inferred that they are very fragile structures. This result is expected since the flocculant molecules are scarce, and therefore the probability of formation of bridges of any type is low. On the contrary, at high doses of flocculant in seawater, the flocs are large, and, in addition, according to their high fractal dimension (between 2 and 2.5), they have a resistant structure. This result is also expected because the greater the number of flocculant molecules, the greater the probability of forming interparticle bridges.

### 3.4. Resistance of Aggregates

The strength of the aggregates is obtained from the temporal evolution of the average size of the flocs when they face changes in the intensity of mixing, following the methodology recently proposed by Jeldres et al. [25]. Here we analyze the impact of flocculation time, flocculant dose, and type of water on the resistance of kaolin aggregates (4% by weight) flocculated with polyacrylamide.

**Figure 6:**
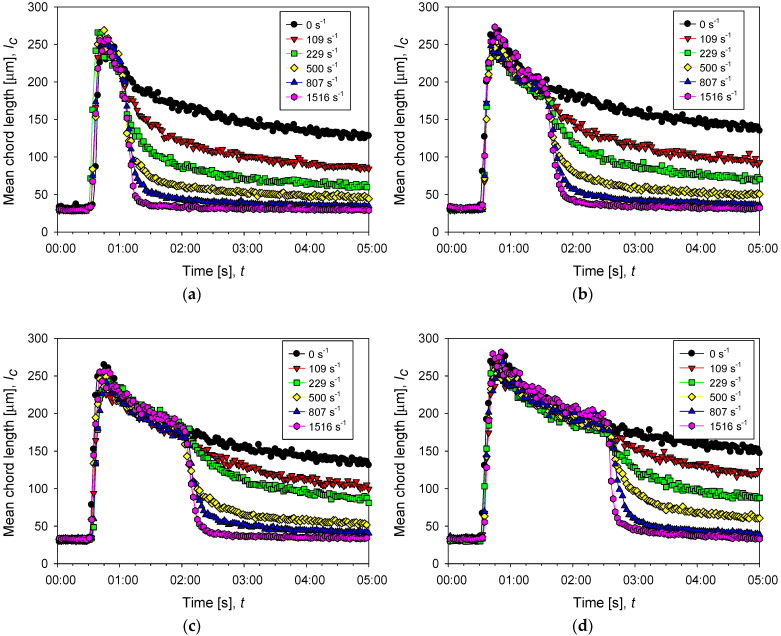
Evolution of the average size of aggregates of kaolin in suspensions with different doses of flocculant in SW and IW at a shear rate of 200 rpm (black dots) and after several increments of shear rate (color dots). The curves with increased shear show three stages, pre-flocculation (primary particles remain free), flocculation (particle size first increases sharply by aggregation and then decreases smoothly with time), and disaggregation (particle size decreases abruptly as shear is increased). (**a**–**c**) correspond to 13, 34, and 89 g/t of flocculant in seawater, respectively, while (**d**) corresponds to 89 g/t of flocculant in industrial water.

Figure 6 shows the change in flocculation kinetics when the system is subjected to increased agitation intensity. The responses are described by fitting the experimental data to Equation (5):

(5) $$y\left(t\right)=y_{0}-A\left(1-e^{-bt}\right)$$

where $y\left(t\right)$ is the average size of the aggregates as a function of time, $y_{0}$ is the average size of the aggregates just before the increment in agitation, b is the decay constant of the aggregate size, and A represents the amplitude of the size reduction of the aggregates, as shown in Equation (6).

(6) $$A=y_{0}-y_{\infty }$$

where $y_{\infty }$ corresponds to the size reached after applying a specific increase in shear rate during an infinite time.

**Figure 7:**
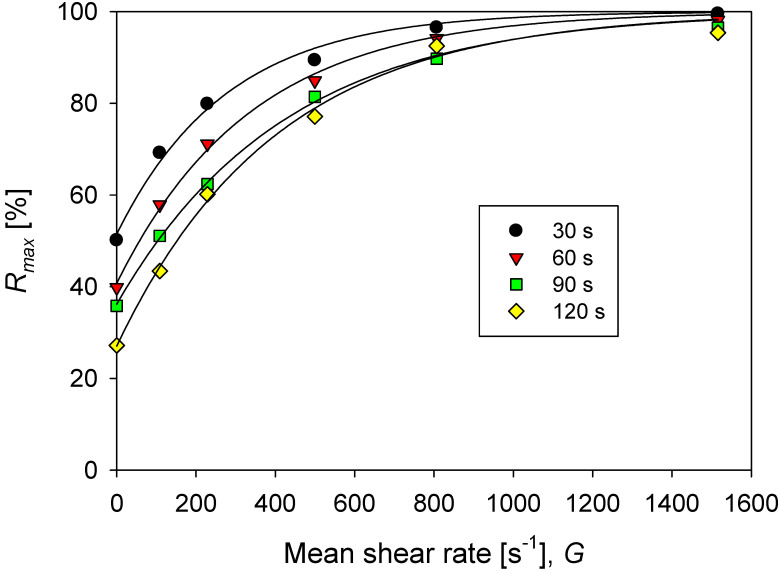
Percentage of maximum rupture (at an infinite time) of aggregates of kaolin in solutions with different doses of flocculant in SW and IW for each increase in agitation from the base of *G* = 273 s^−1^ (200 rpm).

The maximum aggregate rupture at an infinite time for each increment in shear rate (Δ*G*) is estimated from Equation (7).

(7) $$R_{max}=100\times A/\left(y_{0}-d_{0}\right)$$

where $R_{max}$ is the maximum aggregate rupture percent and $d_{0}$ is the average size of the primary particles. The $R_{max}$ values calculated for aggregates at different conditions can be represented by Equation (8), which allows estimating the degree of rupture as a function of the agitation increments (Δ*G*). The results are shown in Figure 7.

(8) $$R_{max}=R_{max,0}+U\left(1-e^{-B\cdot \Delta G}\right)$$

where $R_{max,0}$ is the maximum aggregate rupture percent when there is no disturbance of agitation (constant mean shear rate of 273 s^−1^), *U* is the difference between the maximum aggregate rupture at an infinite shear rate (Δ*G* → ∞) and the rupture degree at the base agitation of 273 s^−1^ (Δ*G* = 0), and *B* is the constant of flocs rupture due to the increase in agitation, whose inverse is considered a characteristic shear rate for the disintegration that should be useful as an indicator of the resistance of aggregates.

After fitting the data to Equation (4), the restriction indicated in Equation (9) is added, which corresponds to the system response when the mixing intensity is very high (Δ*G* → ∞). In this case, a total rupture of the aggregate is considered, giving rise to a suspension of dispersed primary particles.

(9) $$R_{max,0}+U=100\%$$

The value of R=1/B required to obtain $R_{max}-R_{max,0}=63.2\%$ defines the characteristic shear rate or aggregate resistance. The higher the characteristic shear rate, the greater the resistance of the aggregates. In a recent work [25], we show that this simple criterion offers advantages over current methods of estimating the strength of aggregates under increased shearing. We use it to analyze the strength of kaolin aggregates in pulps with different salinities. The strength of aggregates is their opposition to disintegration when shear is increased. The more resistant the aggregate, the lower the fragmentation. The practical impacts are varied, for example, in the design of thickener feedwells in clarification and water recovery operations.

**Figure 8:**
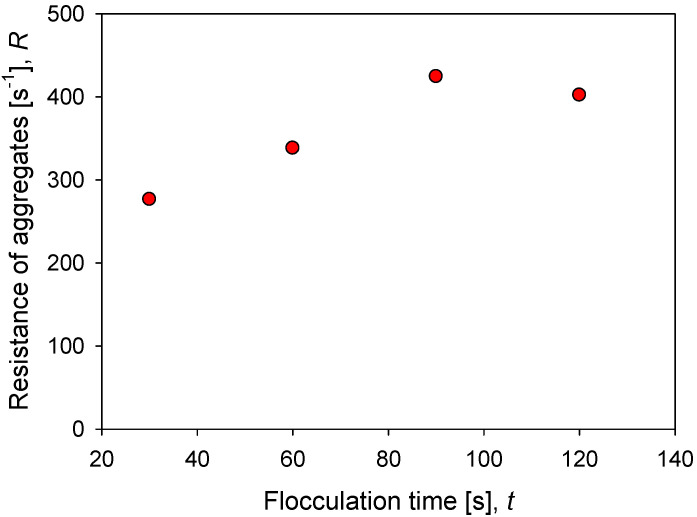
Effect of flocculation time on the resistance of kaolin aggregates (*R*) flocculated in SW with 89 g/t of flocculant.

Figure 8 shows the impact of flocculation time (prior to application of increased agitation) on the strength of kaolin aggregates in seawater at a particular dosage of flocculant. The minimum resistance R for the system and conditions studied is obtained after 30 s of mixing and reaches R = 276 s^−1^. The resistance increases steadily up to 90 s of mixing, reaching a maximum value of R = 424 s^−1^. After this maximum, a longer mixing time leads to a decrease in aggregate strength, R = 400 s^−1^. The smaller structures that are obtained with extended mixing are less prone to breakage and in the case studied here, they dominate the behavior above 100 s of flocculation, a condition in which the aggregates present stable size and structure.

**Figure 9:**
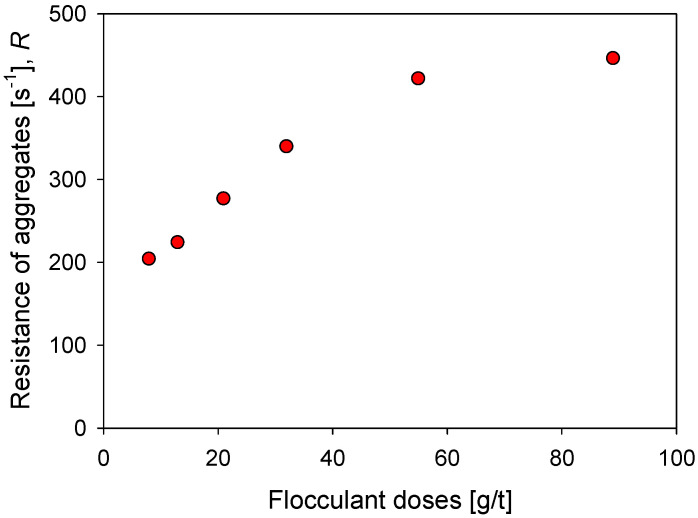
Effect of the flocculant dose on the resistance of kaolin aggregates (*R*) flocculated in SW after 60 s flocculation time.

Figure 9 shows the impact of the flocculant dosage on the strength of kaolin aggregates in seawater after a flocculation time of 60 s. With 8 g/t of flocculant, a minimum resistance *R* of 203 s^−1^ is obtained, which increases monotonically with the dose until it reaches a value slightly higher than R = 400 s^−1^ with 55 g/t of flocculant, and tends to settle after R = 445 s^−1^ with 89 g/t of flocculant. The flocculant dose is a critical aspect in the formation of the aggregates since it determines the number of polymer molecules available in the suspension to join the particles. Therefore, for a fixed surface area, an increase in polymer molecules is expected to generate a greater number of particle–flocculant bonds or a more intricate particle network. This explains the increasing aggregate strength in Figure 9 with flocculant dosages increasing from 8 to 55 g/t. At higher doses, 89 g/t, the resistance does not change significantly despite having a greater number of polymer molecules. In this case, it should be considered that the large aggregates are more prone to breakdown, which limits the value of the resistance.

**Figure 10:**
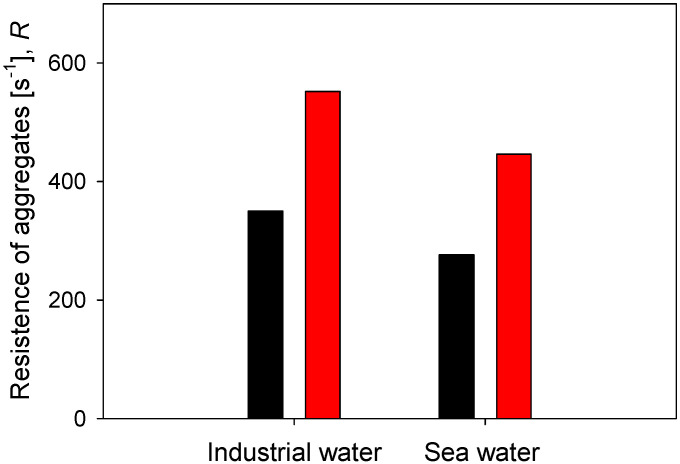
Effect of water type on the resistance of kaolin aggregates (*R*) flocculated at two doses of flocculant after 60 s flocculation time.

Finally, the resistance of flocs in two types of water, industrial water (low salt load) and seawater (high salt load), is compared, considering two doses of flocculant and 60 s of flocculation time. The results are summarized in Figure 10. At any of the flocculant doses tested, the aggregates are more resistant in industrial water than in saltwater. At 21 g/t of flocculant, the resistance is R = 350 s^−1^ in industrial water and only R = 276 s^−1^ in seawater. At 89 g/t of flocculant, the resistance is R = 552 s^−1^ in industrial water and only R = 446 s^−1^ in seawater. Furthermore, the increase of flocculant dose in the same type of water leads to a greater resistance of the aggregates in industrial water than in seawater. When the dose increases from 21 to 89 g/t in industrial water, the resistance increases in 202 s^−1^, while in seawater, it increases only in 170 s^−1^. These results of the effect of salinity on the strength of kaolin aggregates were anticipated by Jeldres et al. recently [25]. The resistance of kaolin aggregates in freshwater without salt reaches a characteristic shear of R = 530 s^−1^, while in saltwater 0.1 M NaCl, the resistance reaches a modest R = 320 s^−1^. These authors observed that an increase in salinity generates aggregates that are more prone to fragmentation due to several factors. For example, changes in the aggregation modes of the primary particles, coiling of the flocculant chains due to a reduced repulsion between anionic functional groups, and changes in the particle–flocculant interactions due to a prevalence of the train configuration in the adsorption mode of the flocculant.

The resistance response of kaolin aggregates to different salinities was somewhat greater in the study by Jeldres et al. [25], whose experiments were carried out at pH 5.5. In contrast, in the present study, the experiments were carried out at pH 8, at which the kaolin particles are mostly anionic. At pH 5.5, in the work of Jeldres et al., kaolin particles in freshwater form aggregates by electrostatic attraction between cationic edges and anionic faces (giving rise to the well-known house-of-cards structure). However, if the ion concentration increases, the electrostatic shielding of the mineral surface reduces the number of edge–face bonds, and face–face type organizations prevail, which are more fragile, and also the efficiency of the flocculant is critically reduced by coiling. A pH of 8 does not favor edge–face bonds that provide resistance to the kaolin aggregates, and thus their resistance is somewhat lower than at pH 5.5. In the presence of seawater and at a pH of 8, on the one hand, salt bridges are activated that contribute to the resistance of the aggregates, and on the other, the flocculant chains are deactivated due to coiling; the resistance of the aggregates is the net effect of these two factors.

## 4. Conclusions

The time evolution of the average size of the kaolin flocs measured when the particle suspension is subjected to incremental shear rates from a predetermined base value, reveals a shear rupture rate characteristic of these aggregates and their formulation. We associate this shear rate with the upper strength limit of the structure of these aggregates. In this way, the effect of flocculation time, flocculant dose, and water quality on the shear strength of kaolin aggregates are studied. The kaolin aggregates are flocculated with a high molecular weight anionic polyelectrolyte at a pH of about 8. In seawater, at a given dose of flocculant, the strength of the aggregates increases with time up to a maximum value; however, at longer times, the resistance decreases until settling at a stable value corresponding to stable aggregates in size and structure. A higher flocculant dosage leads to stronger aggregates due to more particle–polymer bridges, leading to a more resistant particle network. In industrial water with low salt content, the resistance of the kaolin aggregates is higher than in seawater at the same dose of flocculant. Thus, the salt content weakens the resistance of the aggregates and works against the efficiency of the flocculant, corroborating previous studies. The kaolin aggregate rupture strength criterion used in this work is simple, obeys standardized tests that are relatively easy to replicate in the laboratory, and is useful for decision making in the design and operation of water thickeners and clarifiers.

## Figures and Tables

**Table 1:** Ionic concentration of seawater and methods of analysis.

Ion	Concentration (g/L)	Method
${\mathrm{Na}}^{+}$	10.9	Atomic absorption spectrometry
${\mathrm{Mg}}^{2+}$	1.38	Atomic absorption spectrometry
${\mathrm{Ca}}^{2+}$	0.4	Atomic absorption spectrometry
$K^{+}$	0.39	Atomic absorption spectrometry
${\mathrm{Cl}}^{-}$	19.6	Argentometric method
${\mathrm{HCO}}^{3-}$	0.15	Acid–base volumetry

**Table 2:** Data to calculate the mean shear rate.

Parameter	Value	Unit of Measurement
${µ}_{sus}$	0.004	kg/(m·s)
$N_{p}$	0.6	
D	0.08	m
W	0.04	
$\rho_{s}$	2600	kg/$m^{3}$
$\rho_{w}$	1000	kg/$m^{3}$
V	0.25	L

## Data Availability

The data presented in this study are available on request from authors K. Pérez, M. Jeldres, and R.I. Jeldres.
